# Quantitative accuracy of attenuation correction in the Philips Ingenuity TF whole-body PET/MR system: a direct comparison with transmission-based attenuation correction

**DOI:** 10.1007/s10334-012-0328-5

**Published:** 2012-08-26

**Authors:** Georg Schramm, Jens Langner, Frank Hofheinz, Jan Petr, Bettina Beuthien-Baumann, Ivan Platzek, Jörg Steinbach, Jörg Kotzerke, Jörg van den Hoff

**Affiliations:** 1 PET Center, Institute of Radiopharmacy, Helmholtz-Zentrum Dresden-Rossendorf, Dresden, Germany; 2 Department of Nuclear Medicine, University Hospital Carl Gustav Carus, Technische Univserität Dresden, Dresden, Germany; 3 Department of Radiology, University Hospital Carl Gustav Carus, Technische Universität Dresden, Dresden, Germany

**Keywords:** PET/MR, MR-based attenuation correction, PET quantification

## Abstract

**Objective:**

Evaluation of the quantitative accuracy of MR-based attenuation correction (MRAC) in the Philips Ingenuity TF whole-body PET/MR.

**Materials and methods:**

In 13 patients, PET emission data from the PET/MR were reconstructed using two different methods for attenuation correction. In the first reconstruction, the vendor-provided standard MRAC was used. In the second reconstruction, a coregistered transmission-based attenuation map from a second immediately preceding investigation with a stand-alone Siemens ECAT EXACT HR^+^ PET scanner was used (TRAC). The two attenuation maps were compared regarding occurrence of segmentation artifacts in the MRAC procedure. Standard uptake values (SUVs) of multiple VOIs (liver, cerebellum, hot focal structures at various locations in the trunk) were compared between both reconstructed data sets. Furthermore, a voxel-wise intensity correlation analysis of both data sets in the lung and trunk was performed.

**Results:**

VOI averaged SUV differences between MRAC and TRAC were as follows (relative differences, mean ± standard deviation): (+12 ± 6) % cerebellum, (−4 ± 9) % liver, (−2 ± 11) % hot focal structures. The fitted slopes of the voxel-wise correlations in the lung and trunk were 0.87 ± 0.17 and 0.95 ± 0.10 with averaged adjusted *R* ^2^ values of 0.96 and 0.98, respectively. These figures include two instances with partially erroneous lung segmentation due to artifacts in the underlying MR images.

**Conclusion:**

The MR-based attenuation correction implemented on the Philips Ingenuity PET/MR provides reasonable quantitative accuracy. On average, deviations from TRAC-based results are small (on the order of 10 %  or below) across the trunk, but due to interindividual variability of the segmentation quality, deviations of more than 20 %  can occur. Future improvement of the segmentation quality would help to increase the quantitation accuracy further and to reduce the inter-subject variability.

## Introduction

The recent introduction of dedicated clinical whole-body PET/MR systems by [1] and Siemens Healthcare [2] represents an important milestone in the development of combined PET/MR imaging. First systems were installed during 2010 by Philips and Siemens and reached CE approval in 2011.

As discussed, e.g., in Refs. [3–9], the prospects of combined PET/MR in bimodal functional imaging are huge in various clinical fields, notably in oncology, neurology, and cardiology. Compared to PET/CT, PET/MR profits from the superior soft tissue contrast of MRI and offers the option of combining functional PET and MR imaging techniques which go well beyond what is possible with PET/CT [6, 9]. A further obvious advantage is the lack of radiation exposure in MRI examinations.

The actual combination of PET and MRI faced many challenges [6, 9]. One major obstacle was the mutual adverse effects of the PET and MRI hardware and data acquisition/processing electronics and the magnetic field intolerance of the photomultipliers. Philips and Siemens solved these problems by developing the sequential Ingenuity TF PET/MR and the integrated Biograph^TM^mMR PET/MR, respectively. The former system, which was installed at our institute in the fall of 2010, employs established PET and MR technology with suitable modifications while the latter system relies on new detector technology for the PET component.

Another major challenge, which affects all current PET/MR designs equally, is the development of a reliable MR-based attenuation correction (MRAC) for PET image reconstruction. Attenuation correction (AC) is mandatory in order to obtain correct regional PET image contrast and to enable quantitative assessment of regional tracer concentrations and derived parameters such as standard uptake values (SUVs) or tracer kinetic parameters.

Commonly, AC in PET is based on one of the following two methods:

- In stand-alone PET systems, AC is based on a direct measurement of photon attenuation by performing a separate transmission scan (TRAC). For this purpose, one or more suitable radioactive sources are rotated around the body. Based on this measurement and a blank scan the transmitted photon fraction is determined and may be directly used for AC. In the case of whole-body PET measurements with the Siemens ECAT EXACT HR^+^-scanner the AC factors are used to reconstruct a tomographic image of the 511-keV attenuation coefficients. A forward projection of the weighted sum of the reconstructed image itself and a segmented version is finally used for AC [10–13].
- In today’s PET/CT systems, radioactive sources are no longer available. Instead, AC is based on a CT scan. The required transmission map is obtained by bilinear scaling of Hounsfield units [14, 15].

In PET/MR, however, where neither a CT nor a transmission source is available, a different strategy must be used. Hofmann et al. [16] discussed several approaches to MRAC including:

- intensity-based tissue type segmentation and classification of an MR image [17–20]
- atlas-based segmentation techniques [21, 22]

both followed by an assignment of the respective attenuation coefficients according to the tissue type.

Currently, both commercially available whole-body PET/MR systems implement MRAC based on approach (A). While both systems provide attenuation corrected PET images with plausible regional contrast and quantitative parameters such as SUVs, a thorough in vivo evaluation and validation is missing up to now.

Several groups have already investigated the quantitative influence of segmented attenuation maps on reconstructed PET data. Refs. [19] and [23] used segmented attenuation maps based on CT scans and purely simulated data, respectively, whereas Refs. [17, 20, 24] compared data from PET/CT investigations and MRAC reconstructions of several patients where MR-based attenuation maps (MRmaps) were derived from aligned MR scans performed in addition to PET/CT scans. However, no work exists comparing MRAC to TRAC which can be considered the de facto gold standard for attenuation correction. Thus, the aim of our investigation is the evaluation of the quantitative accuracy of MRAC as currently implemented in the Ingenuity PET/MR [17, 20] by a comparison to TRAC.

## Materials and methods

### Data acquisition

In our analysis, we investigated 13 patients who first underwent examination with the stand-alone ECAT EXACT HR^+^ [25] PET scanner (Siemens, Erlangen, Germany) and, subsequently, a second examination with the Ingenuity TF PET/MR (Philips, Cleveland, US). Details of patient demographics are shown in Table 1. For patients 1–9 a whole-body PET protocol was used, whereas patients 10–13 were examined with a head-neck PET protocol. In the two successive PET scans, care was taken to ensure comparable positioning of the patient and to cover a similar axial field of view (FOV).

**Table 1:** Patient demographics

	Clinical indication	Sex	Age (years)	Height (cm)/ weight (kg)	Injected activity (MBq)	Uptake times (min) HR^+^/PETMR	Protocol HR^+^/PETMR	Truncated FOV (cm)
1	Lymphoma	F	51	168/75	301	60/120	WB/WB	6
2	Tonsil Ca	M	62	167/55	372	65/225	WB/WB	4
3	Lymphoma	M	55	167/72	348	65/160	WB/WB	4
4	Lymphoma	M	71	164/60	288	60/170	WB/WB	4
5	Lymphoma	M	27	183/72	354	90/185	WB/WB	8
6	Sarcoma	M	44	176/82	364	55/180	WB/WB	6
7	Lung Ca	M	55	189/93	350	70/210	WB/WB	6
8	Lymphoma	F	28	163/67	320	60/160	WB/WB	4
9	Head-neck Ca	M	60	170/68	345	70/180	WB/WB	6
10	Squamous cell Ca	M	78	163/81	328	50/170	WB/HN	0
11	Squamous cell Ca	F	82	160/75	363	55/165	WB/HN	0
12	Squamous cell Ca	F	79	146/43	292	60/170	WB/HN	0
13	Squamous cell Ca	M	73	168/65	332	135/205	WB/HN	0

Whole-body (WB) and head-neck (HN) PET protocols were used for examination

The first scan was carried out with the HR^+^ stand-alone PET which permits transmission measurements at 511 keV using rotating ^68^Ge/^68^Ga rod sources. The second scan was conducted in the Ingenuity TF PET/MR which combines a modified Philips Gemini TOF PET with a 3 T Achieva MR. The performance of the system components is essentially identical to what is known from the corresponding PET/CT and stand-alone Achieva MR systems [1].

Depending on body weight, the patients were injected between 288 and 372 MBq ^18^F-FDG. After an uptake period of 60 min all patients underwent a routine clinical ^18^F-FDG scan in the HR^+^. In all cases, the acquisition time of the transmission scan was 4 min per bed position. All examinations took place with arms-down and in free-breathing mode. To improve patient comfort, cushions and pillows were positioned below the head and knees of the patients. After completion of the first examination all patients left the patient bed and stayed in a waiting room for about 60 min. Subsequently, they underwent the PET/MR examination which started, on average, about 180 min p.i. and consisted of:

1. a fast 3D T1-weighted gradient-echo MR sequence (atMR) used for MRmap computation,
2. the PET emission acquisition, and
3. (optional) diagnostic MR sequences.

Throughout the entire examination the patients remained in a fixed position on the patient bed which was moved automatically between the MR and PET gantries. The parameters of the atMR scan were: flip angle 10°, TE 2.3 ms ,TR 4 ms, minimal water-fat shift, no water or fat saturation and voxel size 3 mm × 3 mm × 6 mm. 12 cm 3D stacks were acquired with 12 mm overlap covering a total transaxial FOV of 60 cm. The acquisition time per stack was 16 s. In all cases, the Q-Body coil was used for the atMR image acquisition. For the head-neck investigations, an NV 16 head-neck coil was additionally connected but only used for acquisition of diagnostic MR sequences following the PET scan. Again, all patients were examined in arms-down and free-breathing mode.

Subsequent to the atMR, PET emission data were acquired for 2 min and 6 min per bed position in the whole-body and head-neck investigations, respectively.

### Image reconstruction

**Fig. 1:**
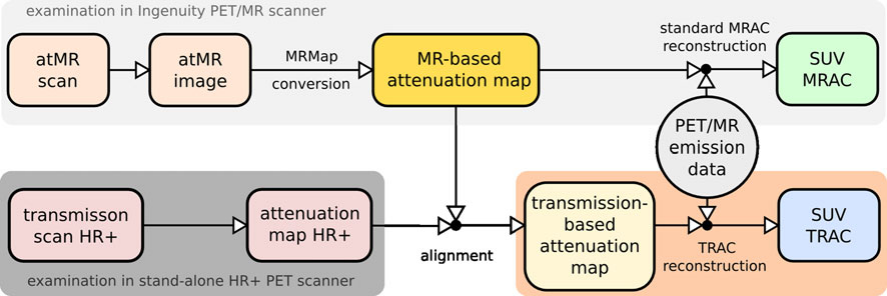
Scheme of the two reconstructions of the PET/MR emission data using MR-based attenuation correction and an external transmission-based attenuation correction. Further details are given in the text

Figure 1 shows the work-flow of our analysis consisting of two separate reconstructions of the PET/MR emission data. First, we executed the vendor-provided standard MRAC reconstruction, shown in the top branch. The MRmap is derived by tissue type segmentation and classification of the atMR images acquired in the atMR sequence (MRmap conversion) [17, 20]. The conversion algorithm segments the atMR image into air, lung and soft tissue, adjusts the spatial resolution (Gaussian filter, 6 mm FWHM), and assigns linear attenuation coefficients of 0, 0.022, and 0.096 cm^−1^, respectively. For both types of protocols (whole-body and head-neck), the MRmap conversion algorithm works in the same way. The result of the standard reconstruction using MRAC is a first PET image volume called PET_MRAC_ in the following.

As shown in the lower branch of Fig. 1, we performed a second reconstruction of the PET/MR emission data using the transmission scans from the HR^+^ examinations as external information about photon attenuation (TRAC).

For this purpose, we first removed information about attenuation of all structural materials (bed and coils) from the TRmaps and MRmaps. In case of the TRmaps, this was done by subtracting a separate transmission scan of the HR^+^ bed. In the MRmaps we removed the existing (vendor-provided) attenuation templates for patient bed and respective coils.

Secondly, we coregistered the obtained TRmaps to the corresponding MRmaps. Coregistration was performed with the software ROVER (ABX GmbH, Radeberg, Germany) using a rigid transformation to maximize the mutual information between both data sets [26]. Since the head and leg supports are rather different in the HR^+^ and PET/MR systems, these parts of the image volumes were excluded from the rigid coregistration. In the cases of the head-neck investigations automatic coregistration of the heads did not work because of the different inclination of the head/neck relative to the thorax. Instead, we manually coregistered the brain region of the HR^+^ PET image to the corresponding region in the atMR image volume. Subsequently, we applied the resulting rigid transformation to the transmission-based attenuation map of the HR^+^. Correct alignment relative to the atMR was finally double-checked by a further visual inspection.

After conversion of the attenuation maps to the proprietary Philips “.img” file format, templates for patient bed and the respective coils were added to the TRmaps. The PET image volume resulting from this second reconstruction will be called PET_TRAC_ in the following. In the absence of possible errors caused by residual registration mismatch between the TRmap and the emission data set, we consider PET_TRAC_ as the de facto gold standard against which the PET_MRAC_ data set can be evaluated.

### Image analysis

For the analysis we first outlined a number of 3D regions (volumes of interest: VOIs) in PET_MRAC_ and PET_TRAC_ for which individual voxel values as well as summary parameters were compared.

**Fig. 2:**
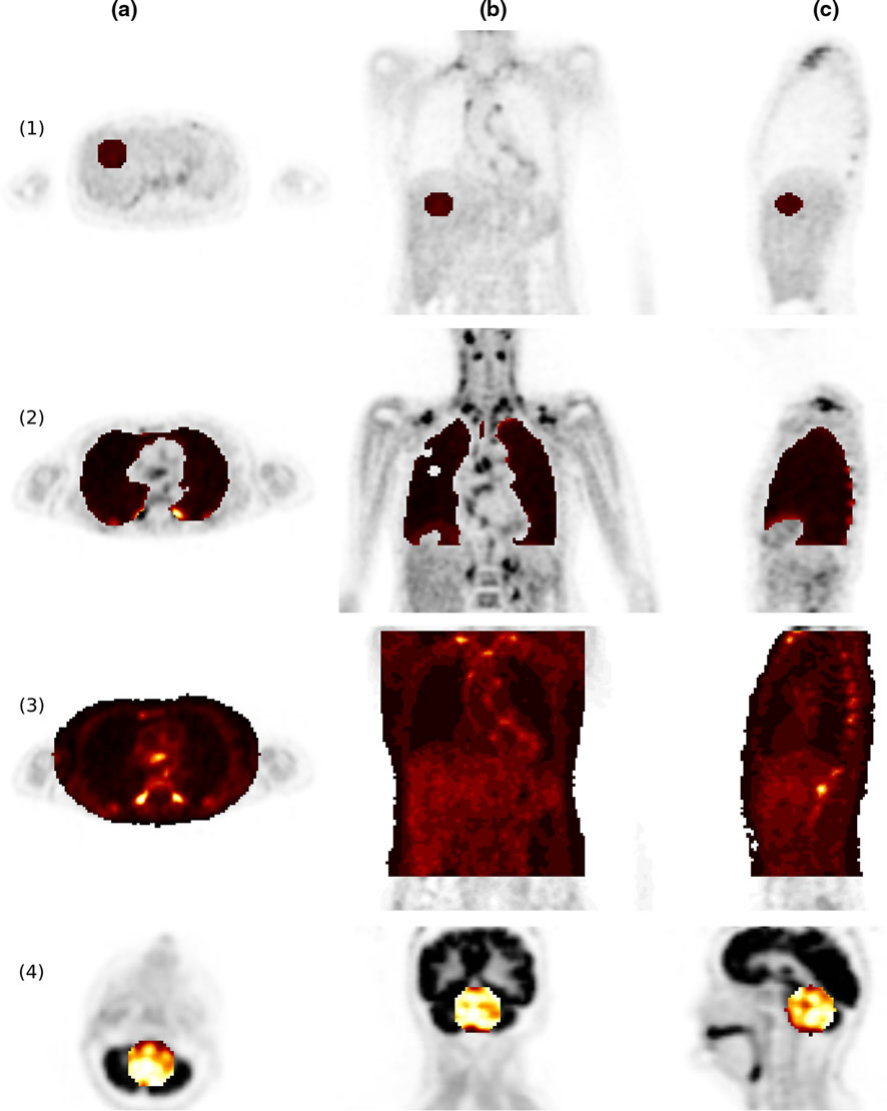
Representative transaxial (**a**), coronal (**b**) and sagittal (**c**) views of VOI definition in liver (*1*), lung (*2*), trunk (*3*) and cerebellum (*4*)

In the whole-body data sets we manually placed VOIs in the liver and the lung. Additionally several hot focal structures above the liver were delineated using the automatic VOI delineation method implemented in the ROVER software [27] (ABX, Radeberg, Germany) which uses adaptive thresholding for volumetrically correct VOI determination [28]. The body region below the liver was excluded from the comparison because of possible significant peristaltic movement between the two examinations. In the head-neck investigations, we placed a single VOI in the cerebellum. Examples for all VOIs, except for the hot focal structures, are shown in Fig. 2.

In the liver, cerebellum and hot focal structure VOIs we investigated the absolute and relative differences

1 $$ \Updelta\hbox{SUV}_{\rm abs} = \hbox{SUV}_{\rm MRAC} - \hbox{SUV}_{\rm TRAC} $$

2 $$ \Updelta\hbox{SUV}_{\rm rel} = \frac{\Updelta\hbox{SUV}_{\rm abs}} {\hbox{SUV}_{\rm TRAC}} $$

of the SUVs between the two PET image volumes. In each VOI, the deviation of the maximum ($\Updelta \hbox{SUV}_{\rm max}$) and the mean values ($\Updelta \hbox{SUV}_{\rm mean}$) was determined.

We furthermore performed a linear regression analysis of the SUV correlation for all voxels in the complete lung and trunk (see Fig. 2). VOI delineation was performed using manual thresholding in the atMR images (lung) and PET images (trunk), respectively. A straight line through the origin was fitted to the voxel data according to

3 $$ \hbox{SUV}_{\rm MRAC} = m \cdot \hbox{SUV}_{\rm TRAC}. $$

The goodness of fit was assessed using adjusted *R* ^2^-values.

## Results

### Visual comparison of the attenuation maps and reconstructed PET volumes

**Fig. 3:**
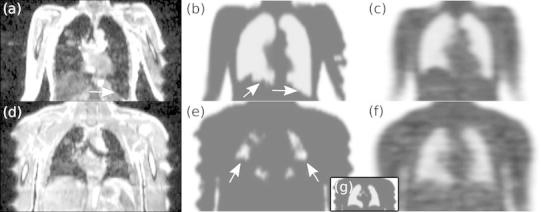
Two instances of incorrect lung segmentation. Corresponding slices of atMR (*left*), MRmap (*middle*), and TRmap (*right*) are shown for patient 1 (**a**–**c**) and 6 (**d**–**f**), respectively. The inset (**g**) displays the much improved lung segmentation in the latter patient after adjusting the relevant parameter of the segmentation algorithm

Figure 3 shows corresponding coronal slices of the atMR image, the MRmap and the TRmap of patients 1 and 6. Truncation artifacts in the atMR and MRmap are clearly visible. These artifacts are caused by the reduced trans-axial FOV of the MR which is approximately 15 cm smaller than that of the PET scanner. A vendor-provided protocol for compensation of truncation artifacts based on a segmentation of a non-attenuation-corrected PET image exists which works reasonably well in general but imperfectly in selected cases. Therefore, and because the truncation compensation option is not used in our clinical routine, we did not use it in the present investigation.

In the two whole-body investigations shown in Fig. 3 we also observed that lung segmentation in the atMR did not work properly. One case (top row) shows abnormally low signal intensity in the areas below both lungs which leads to an erroneously segmented lung extending too far in the caudal direction. In the second case (bottom row), lung segmentation failed more severely when using the default parameter settings of the segmentation procedure. In this case, the MRmap shows several small cavities erroneously identified as air rather than lung tissue. The reason for this segmentation failure is the presence of pronounced heart motion artifacts in the atMR. The decreased image contrast between lung and soft tissue regions leads to the observed misinterpretation of the atMR by the conversion algorithm. In Fig. 3g the segmentation was repeated with different parameter settings which shows that segmentation failure can be avoided by adjusting the relevant parameters of the algorithm. Since our intention was the evaluation of the current default MRAC procedure the erroneous segmentation (e) was used in our further analysis.

**Fig. 4:**
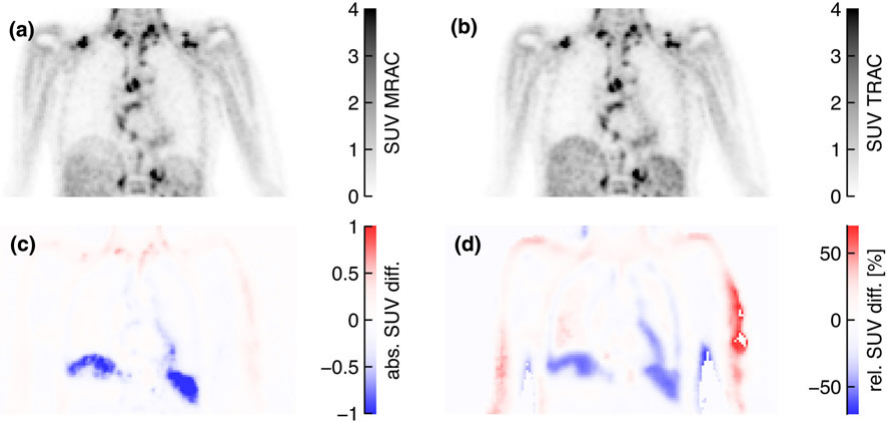
Corresponding coronal PET_MRAC_ (**a**) and PET_TRAC_ (**b**) slices of patient 1. The absolute and relative SUV differences according to Eqs. (1) and (2) are shown in (**c**) and (**d**), respectively. In order to improve visibility of the relevant differences, some spurious voxels in the left arm are suppressed in image (**d**) by choosing a suitable upper threshold

Figure 4 shows corresponding coronal PET_MRAC_ and PET_TRAC_ slices as well as the resulting absolute and relative SUV differences for patient 1. Rather large negative deviations are found in the liver dome and the spleen. Large positive deviations are observed along the arms, which are due to the slightly different positioning of the arms during the PET and PET/MR examinations.

**Fig. 5:**
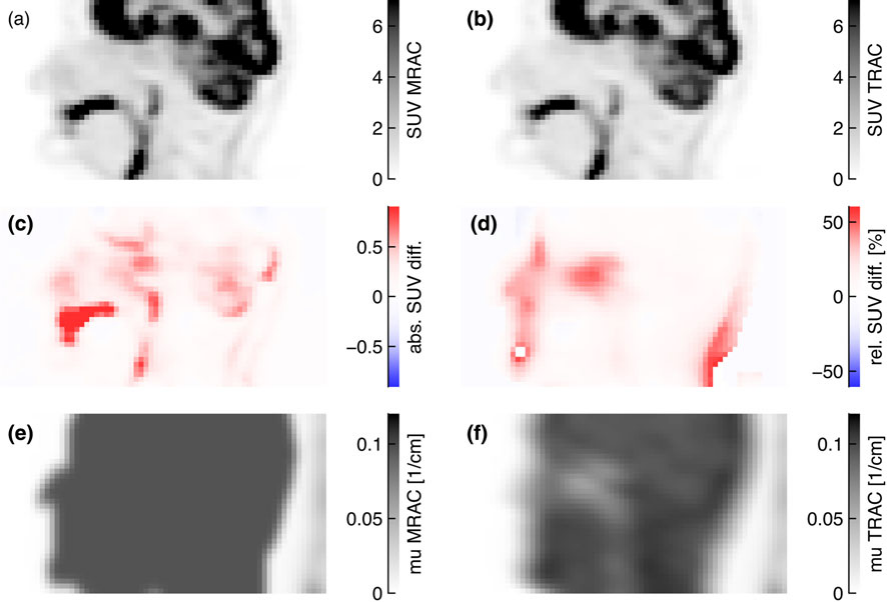
Same as Fig. 4 for a sagittal slice of patient 11. In order to improve visibility of the relevant differences, some spurious voxels in the chin are suppressed in image (**d**) by choosing a suitable upper threshold. Subfigures (**e**) and (**f**) show the corresponding slices of the MRmap and TRmap, respectively

Figure 5 shows the same quantities as Fig. 4 for patient 11 (head-neck investigation). A pronounced relative increase of SUV_MRAC_ can be seen in the face and neck contour (up to +100 %) and in the nasal cavities (+40 %). The former one results from an imperfect alignment of the MRmap and TRmap, whereas the latter is due to the non-segmentation of the air-filled nasal cavities.

### Comparison of reconstructed SUVs in different VOIs

**Fig. 6:**
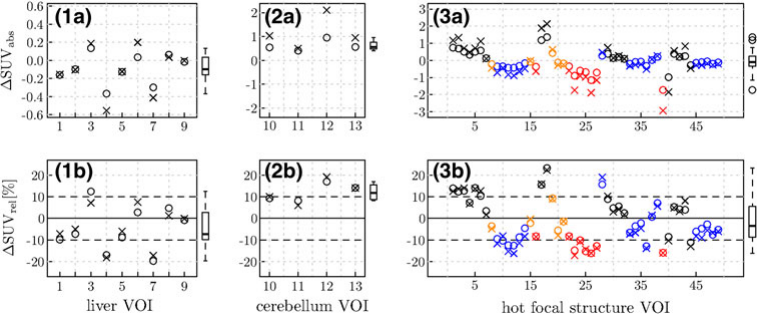
Absolute (*top*) and relative (*bottom*) SUV differences between PET_MRAC_ and PET_TRAC_ in the liver (1), the cerebellum (2), and several hot focal structures (3) located in the lung and mediastinum (*red*), in the thoracic spine (*blue*), in the liver and kidneys (*orange*) and in the clavicular region (*black*), respectively. *Crosses* indicate SUV_max_, *circles* SUV_mean_ deviations in the respective VOIs. The* horizontal dashed lines* show ±10 % deviations. The* boxplots* in the right margins of the plots represent the resulting distributions of the respective SUV_mean_ deviations

Figure 6 and Table 2 show and summarize the absolute and relative SUV differences between PET_MRAC_ and PET_TRAC_ calculated according to Eqs. (1) and (2) in the liver, cerebellum, and hot focal structure VOIs, respectively. Only patients 4 and 7 exhibit SUV_max_ deviations above 10 % in the liver VOI. These data sets show discrepancies regarding the axial extension of the lung between MRmaps and TRmaps (larger in the MRmaps).

**Table 2:** Relative differences (mean ± standard deviation) between SUV_MRAC_ and SUV_TRAC_ in the liver, the cerebellum and several hot focal structure VOIs

VOI	number	$\Updelta \hbox{SUV}_{\rm rel,max}$ [%]			$\Updelta \hbox{SUV}_{\rm rel,mean}$ [%]		
		Mean ± SD	Median	Range	Mean ± SD	Median	Range
Liver	9	−4 ± 9	−5	−18 to 8	−5 ± 10	−7	−20 to 12
Cerebellum	4	12 ± 6	12	6 to 19	12 ± 4	12	8 to 17
Hot focal structures							
Total	49	−2 ± 11	−6	−17 to 22	−1 ± 10	−4	−16 to 23
Lung and mediastinum	8	−13 ± 3	−14	−17 to −9	−13 ± 3	−14	−16 to −8
Thoracic spine	18	−6 ± 8	−8	−16 to 19	−5 ± 7	−6	−12 to 15
Clavicular region	18	7 ± 9	7	−13 to 2	7 ± 8	6	−11 to 2
Liver and kidney	5	−1 ± 6	−1	−8 to 9	−1 ± 6	−2	−6 to 9

In the four head-neck investigations, a systematic overestimation of the SUV_max_ of 12 % is observed.

In the analysis of the 49 hot focal structures, we found a mean SUV_max_ deviation of −2 % with a standard deviation of 11 %. The maximum overestimation was 22 % in brown fatty tissue supraclavicular near the body surface and the maximum underestimation was −17 % in a lesion near the lung boundary.

### Voxel-by-voxel correlation analysis

**Fig. 7:**
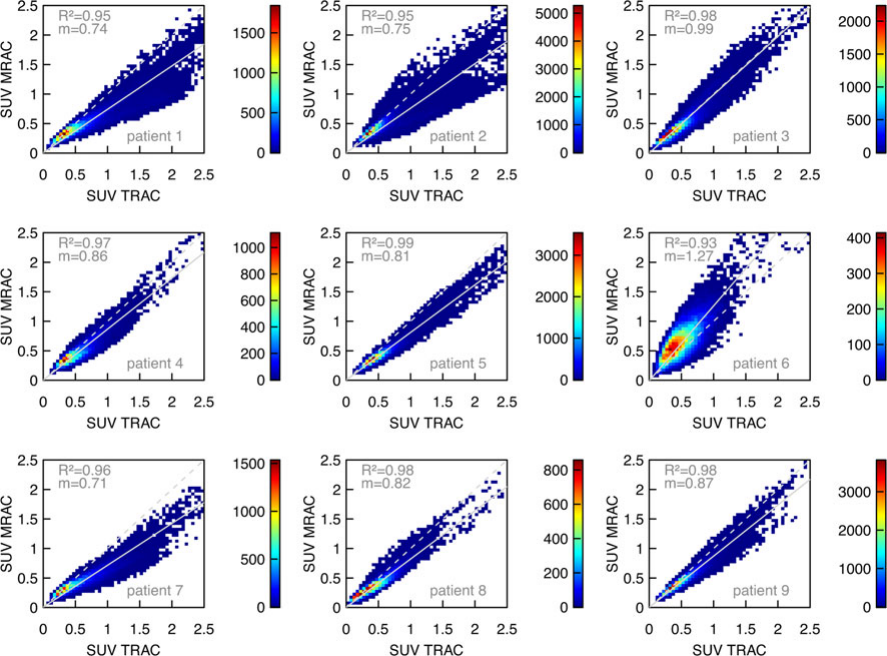
Voxel intensity (SUV) correlation between PET_MRAC_ and PET_TRAC_ in the lungs of patients 1–9. The correlations are displayed as 2D histograms of 50 × 50 bins with *color-coded frequencies*. The* dashed line* indicates the line of identity, the* solid line* represents the best fit of Eq. (3) to the data. The adjusted *R* ^2^ and slope *m* of the fitted straight line through the origin are specified in the plots

**Fig. 8:**
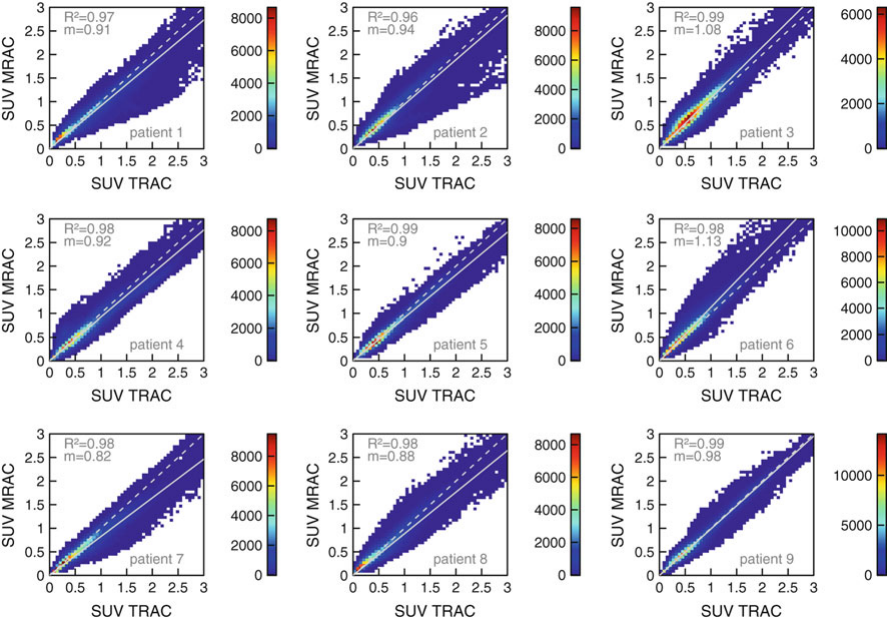
Same as Fig. 7 for all voxels in the trunk of patients 1–9

Figures 7 and 8 display the voxel intensity (SUV) correlation between PET_MRAC_ and PET_TRAC_ in the lungs and in the trunk of patients 1–9, respectively. The results of the fitted slopes are summarized in Table 3. Except in one case, PET_MRAC_ yields lower values in the lung than PET_TRAC_. In patient 6, the SUV_MRAC_ is higher than SUV_TRAC_ while the degree of linear correlation is decreased, exhibiting a rather low adjusted *R* ^2^ of 0.93. As shown in Fig. 3e, these discrepancies are caused by an incorrect lung segmentation in the MRmap generation. By comparing the attenuation coefficients of the lung in both attenuation maps, we found that the assigned value in the MRmaps is approximately 10 % lower than the measured one in the TRmaps. As observed in the lung, SUV_MRAC_ in the trunk is on average lower than SUV_TRAC_ for most patients (with the notable exception of patient 6). However, the bias is distinctly smaller than in the lung alone.

**Table 3:** Results for the slope *m* (mean ± standard deviation) and the averaged adjusted *R* ^2^-value of the correlation analysis between SUV_MRAC_ and SUV_TRAC_ in the lung and trunk according to Eq. (3)

VOI	fitted slope *m* of correlation			*R* ^2^
	mean ± SD	median	range	mean
Lung	0.87 ± 0.17	0.82	0.71–1.27	0.96
Trunk	0.95 ± 0.10	0.92	0.82–1.13	0.98

## Discussion

In general, the vendor implementation of MRAC for the Ingenuity PET/MR system assuming a 3-class tissue separation (air, lung and soft tissue) provides reasonable accuracy with respect to SUV-based quantification. The MRAC corrected PET emission data are quantitatively in agreement with those obtained using TRAC for the same PET emission data within an error margin of about 10–20 % as shown in Fig. 6b and Table 2. However, the exact magnitude of the local deviations between MRAC and TRAC is dependent on the investigated patient and of course also organ specific. The same holds true for the influence of potential segmentation errors (which mostly concern the lung and its vicinity).

In the cerebellum VOIs, we observed a small systematic difference of 12 % between PET_MRAC_ and PET_TRAC_, while otherwise the average differences remained insignificant (i.e. were much smaller than the inter-subject variability).

The probable explanation of the observed SUV bias in the cerebellum is the fact that the air space in the nasal cavity is erroneously treated as soft tissue by the MRAC algorithm. Hence, the attenuation for emission events detected along lines of response crossing the cerebellum and the oral and nasal cavities is overestimated leading to an overcorrection and artificial increase in the reconstructed SUVs in the transaxial planes around the cavities. As the small observed inter-subject variability (4 %) demonstrates, this effect is fairly constant across patients.

In the other investigated regions, the inter-subject variability of the observed differences is modest and amounts to a standard deviation of 10 percentage points in the liver and in the different hot focal structures. The total range of observed deviations exceeds 20 % only in one case.

Closer inspection of the most pronounced deviations revealed that the corresponding VOIs are located at tissue borders (e.g. at the lung border) where linear attenuation coefficients in the MRmaps and TRmaps are not completely concordant due to their different resolution. The observed deviations in these cases are thus at least partly due to the mismatch between the spatial resolution of the external TRmap (ca. 10 mm) and of the MRmap (ca. 6 mm).

In patient 7, where we found the maximum SUV deviation in the liver VOI (−18 %), we observed a larger anterior-posterior extension of the segmented lung in the MRmap than in the TRmap. After manual editing the MRmap, enforcing concordance with the lung extension in the TRmap, the SUV difference dropped to −10 %. The larger anterior-posterior extension can be explained by the different positioning of the legs which can cause different positions of the lumbar spine and the diaphragm in both scans.

The voxel-based intensity correlation analysis shown in Figs. 7 and 8 and Table 3 demonstrates a satisfactory linear correlation between the SUVs from PET_MRAC_ and PET_TRAC_ data. However, as can be seen in Fig. 7, the SUVs in the lung are systematically underestimated in PET_MRAC_. A similar systematic deviation of the reconstructed SUVs is observed in the analyzed hot focal structure VOIs located in the lung and the mediastinum (see Fig. 6; Table 2). This observation can be explained by taking into account that the attenuation coefficient assigned to lung tissue in the MRmaps is about 10 % lower than the corresponding value in the TRmaps. A corresponding increase of this value would lead to a decrease or removal of the currently observed systematic underestimate. We presume that the ultimate cause of this apparently incorrect attenuation coefficient are residual uncertainties involved in the rescaling of Hounsfield units in CT-based attenuation measurements to 511 keV [15, 29].

Regarding the whole trunk (including the lung) we observed only a very small average reduction of SUV_MRAC_ versus SUV_TRAC_ (see Fig. 8; Table 3), which is presumably due to the contribution from lung voxels to the correlation. More important, the inter-subject variability amounts to a standard deviation of 10 percentage points and a range from −18 to +13 percentage points regarding the slope of the best straight line fit through the origin. This translates into corresponding uncertainties to be expected for reasonably sized VOIs in this body region.

Overall, our findings are consistent with earlier evaluations of segmented AC for PET as presented in Refs. [19, 23]. However, this is the first study comparing the performance of MRAC directly against a transmission measurement at the relevant photon energy of 511 keV. While the usability of CT-based attenuation correction for quantitative PET is unquestionable, it is well known that this approach has certain limitations and weaknesses stemming from residual uncertainties in the correct rescaling of Hounsfield units [29], resolution mismatch between PET and CT and its handling, and motion artifacts [30]. Transmission measurements with radioactive sources have different issues, notably the reduced statistical accuracy and low resolution of the measurements but do not suffer from most of the other problems of CT-based attenuation correction. Therefore, we consider transmission measurements still as the most accurate available option for attenuation correction in PET. Our results are thus complementary to those obtained previously in previous MRAC evaluations against CT-based attenuation correction.

Our study, nevertheless, has certain limitations which should be considered. The main drawback of our method is the fact that the transmission measurement was performed in a different system, requiring repositioning of the patients in the PET/MR. Consequently, proper coregistration of both attenuation maps is not guaranteed and has to be performed by suitable means. Since the positioning of the arms, legs, and—especially—of the head was slightly different in both PET systems because of different patient beds, head rests and cushions), a rigid transformation is not sufficient for coregistration of the whole data sets. Coregistration therefore was performed independently for the trunk and the head neck area of the image volumes. Minor residual coregistration mismatch persisted nevertheless to some extent, e.g. in the arms. Moreover, the fact that the patients did not stay on the same patient bed and that a certain time elapsed between both scans implies the possibility of organ movement in the abdomen which would not be accounted for completely by the coregistration. For instance, as observed in three cases, differences in the shape of the lumbar spine caused by the use of leg cushions caused a small displacement of the diaphragm leading to different expansions of the lung in the caudal direction during the examinations. While we visually verified that quality of the coregistration was satisfactory, residual mismatch can be expected to exist and contribute to part of the observed differences. However, since regional variation of the attenuation coefficient in the abdomen is rather small (and good coregistration of the lungs is easy to achieve and verify) we do not expect that coregistration mismatch is a source of serious error in this study.

Another limitation is the lower spatial resolution of the TRmaps compared to the MRmaps. This might cause artifacts at tissue boundaries as seen to some extent at the boundaries of the lungs. Moreover, the segmentation process of the TRmaps used in the whole-body protocols of the HR^+^-scanner to reduce noise lowers the attenuation coefficients of structures with high attenuation values such as bone. However, as shown in [11] the difference in the quantitative accuracy between segmented and non-segmented TRAC is small.

A further limitation of this study is the fact that truncation artifacts were not corrected for in order to replicate our standard clinical protocols. As explained in the methods section the aim of this study was the evaluation of the vendor-provided standard procedure which currently does not use the optional truncation compensation. Hence, the observed underestimation of the SUVs in the trunk and lung includes the underestimation caused by the truncation and, thus, can be considered as a worst case estimation. In order to estimate the quantitative influence of truncation artifacts on the reconstructed SUVs, we performed an additional reconstruction of patient 3, where we manually corrected the attenuation coefficients in the truncated arms in the MRmap. In comparison with the PET volume reconstructed with the truncated MRmap, we found that 80 % of the voxels in the trunk showed deviations of less than ± 5 % with an average deviation of −3 %. Since the size of truncated areas in all patients of this study was approximately the same, similar deviations can be expected in the other cases. Thus, the influence of the truncation artifacts on the reconstructed SUVs is far less serious than erroneous lung segmentation. Nevertheless, automated truncation compensation is desirable and one of the fields where improvements can be expected in the near future.

## Conclusion

The MR-based attenuation correction implemented on the Philips Ingenuity PET/MR provides reasonable quantitative accuracy which is in accordance with transmission-based attenuation correction within an error margin of about 10–20 %. There is some room for improvement regarding the MR sequences and the segmentation algorithm used, whose sporadic partial failure currently is responsible for most observed deviations above 10 %. Currently, segmentation results should be verified in each patient investigation in order to eliminate this source of quantitation error. A further issue is the observed bias in lung SUVs of 13 % on average, which is caused by the value used for the linear attenuation coefficient in the lung. Suitability of this value should be reconsidered. Despite the present limitations, we consider the investigated MRAC algorithm adequate for the usual quantitation tasks in whole-body PET.
